# Quantification of glutathione in the human brain by MR spectroscopy at 3 Tesla: Comparison of PRESS and MEGA‐PRESS

**DOI:** 10.1002/mrm.26532

**Published:** 2016-10-31

**Authors:** Faezeh Sanaei Nezhad, Adriana Anton, Laura M. Parkes, Bill Deakin, Stephen R. Williams

**Affiliations:** ^1^ Centre for Imaging Science Manchester Academic Health Science Centre, University of Manchester Manchester United Kingdom; ^2^ Neuroscience and Psychiatry Unit Manchester Academic Health Science Centre, University of Manchester Manchester United Kingdom

**Keywords:** glutathione, PRESS, MEGA‐PRESS, quantification

## Abstract

**Purpose:**

Glutathione (GSH) is an important intracellular antioxidant in the brain. A number of studies report its measurement by localized ^1^H spectroscopy using PRESS and STEAM. This study evaluates the reliability and accuracy of GSH measurements from PRESS at 3 Tesla (T) and compares the results to those obtained with MEGA‐PRESS.

**Methods:**

Phantoms containing brain metabolites, identical except for variable GSH concentration between 0 and 24 mM, were scanned using PRESS (echo time (TE) = 35 ms) and MEGA‐PRESS (optimized TE = 130 ms) at 3 T. Spectra of the anterior cingulate cortex and occipital cortex in seven healthy volunteers were also acquired.

**Results:**

Phantom GSH concentrations from 0 to 3mM were unreliably quantified using PRESS, although at 4 mM and above there was a linear relationship between measured and true concentrations (R^2^ = 0.99). Using MEGA‐PRESS, there was no signal detected at 0 mM GSH, plus a linear relationship (R^2^ = 0.99) over the full range from 0–24 mM. In brain, concentrations calculated from MEGA‐PRESS and PRESS were significantly different in occipital cortex (*P* < 0.001). Moreover, only MEGA‐PRESS reported significant differences in GSH between the two brain regions (*P* = 0.003).

**Conclusion:**

Due to uncertainties in GSH quantification raised by the study, the authors conclude that physiological concentrations (<4 mM) of GSH cannot be reliably quantified from PRESS (TE = 35 ms) spectra at 3 T. Magn Reson Med 78:1257–1266, 2017. © 2016 The Authors Magnetic Resonance in Medicine published by Wiley Periodicals, Inc. on behalf of International Society for Magnetic Resonance in Medicine. This is an open access article under the terms of the Creative Commons Attribution License, which permits use, distribution and reproduction in any medium, provided the original work is properly cited.

## INTRODUCTION

Glutathione (GSH) is a tripeptide consisting of glutamate, cysteine, and glycine present in cells at millimolar concentrations. It plays a role in cell signaling, protein function, gene expression, cell differentiation, and cell proliferation in the brain. Most importantly, it acts as an intracellular antioxidant, protecting against reactive oxygen species in the brain 1. GSH content differs between brain regions, which may reflect regional variation in the availability of GSH for cellular and extracellular functions. Forebrain and cortex appear to have the highest GSH content, followed by cerebellum, hippocampus, striatum, and substantia nigra 2, 3. Disorders of GSH metabolism, causing a decrease in its concentration, highly correlate with increased levels of oxidative stress generated by reactive oxygen species in the brain 4. Therefore, accurate and reliable estimation of GSH concentration in vivo is of great interest and clinical significance.

Detection of GSH in vivo in the human brain can be achieved using proton magnetic resonance spectroscopy (^1^H MRS). The ^1^H MRS detection limit in vivo of approximately 100 µM greatly simplifies the spectral appearance of a human brain; still, the relatively short echo time (TE) spectrum (TE = 20 ms) contains more than 15 different metabolite signatures 5. The signal from GSH is obscured by creatine, aspartate, glutamate, glutamine, N‐acetylaspartate and γ‐aminobutyric acid 6, such that no single signal peak or combination of peaks in the spectrum can be unambiguously assigned to GSH. The fact that the GSH signal is not visually identifiable in PRESS and STEAM spectra renders its quantification solely dependent on solving a complex mathematical equation: fitting the signal to a model function of spectroscopic components in either the time or frequency domain 7, 8, 9 and assuming that the GSH signal lies in the spectra. This estimation is prone to errors and bias: The algorithm will often find a solution, which may be stable and consistent (low Cramer‐Rao lower bounds (CRLB)) and within the expected range, but can still be inaccurate. Another challenge is the lack of consensus about the spectroscopic properties of GSH glycine signal 10, which directly affects the prior knowledge required for model‐based fitting. Regardless of these difficulties in measuring GSH, there have been reports of GSH quantification from PRESS and STEAM spectra 11, 12, 13, 14, 15, 16, 17.

To overcome the quantification uncertainties for low concentration (<4 mM) metabolites such as GSH and γ‐aminobutyric acid (GABA), spectral editing techniques such as J‐difference editing 18 and multiple quantum coherence editing have been described 19, 20. J‐difference spectroscopy differs from multiple quantum coherence editing, as the latter is done in a single acquisition and is less susceptible to movement and spectrometer instability. However, J‐difference methods provide single resonances within the subspectra, which can be used for accurate phase and frequency correction and are now the method of choice for estimating GABA in the human brain 21, 22, 23, 24, 25.

The J‐difference‐edited ^1^H MRS pulse sequence MEGA‐PRESS (MEscher–GArwood‐Point RESolved Spectroscopy) has been introduced to measure GSH 18, based on quantification of the visually detectable GSH cysteine β‐proton resonating at approximately 2.95 ppm. This measurement method has successfully been applied to quantify GSH in a number of studies 26, 27, 28, 29, 30.

The objective of this work was to evaluate the accuracy and precision of GSH quantification from two different methods used to acquire spectra from the human brain at 3 Tesla (T), namely, PRESS (TE = 35 ms) and MEGA‐PRESS. We performed simulations and took preliminary measurements on phantoms and human subjects to confirm the optimum TE for GSH detection by MEGA‐PRESS, as a variety of echo times (TE = 68–136 ms) 26, 27, 28, 29 have been reported. The primary outcome of our study is the comparison of the performance of the sequences in GSH quantification on a series of brain‐mimicking phantoms, containing variable amounts of GSH (0–24 mM) and a constant composition of other brain metabolites at physiological concentrations. A complementary outcome is derived from studying seven healthy volunteers, in whom we compared the performance of the two methods in measuring GSH in two regions of the brain.

## METHODS

All acquisitions, in vitro and in vivo, were performed on a 3T MR scanner (Philips Achieva, Best, the Netherlands) using a body coil for transmission and an eight‐channel head coil for signal reception.

### MEGA‐PRESS TE Optimization from GSH/Ace Phantom and Simulation

A phantom containing 25 mM GSH and 25 mM sodium acetate (Ace) was prepared in phosphate buffer (25 mM KH_2_PO_4_; 25 mM K_2_HPO_4_) and adjusted to pH 7.0. All chemicals were from Sigma‐Aldrich (Dorset, United Kingdom). Phantom temperature was kept at 21 ºC for data acquisition. This phantom was used to study the effect of TE on the GSH signal from MEGA‐PRESS. The two pulses of MEGA‐PRESS were set to the frequency of the cysteine resonance at 4.56 ppm (coupled with the detected signal at 2.95 ppm) and at 1.44 ppm symmetrically disposed at approximately 2.95 ppm. The pulses were Gaussian with 14 ms duration and 106 Hz bandwidth. TE was varied from 70–240 ms in 10 ms steps. The first PRESS echo time TE_1_ was fixed at 12.6 ms, and the second echo duration TE_2_ was adjusted to change the total TE (=TE_1_+TE_2_). The spectra were acquired in blocks of four averages when the MEGA pulse was set at 4.56 ppm (MEGA‐on), referred to as a single dynamic, followed by four averages of MEGA pulse set at 1.44 ppm (MEGA‐off). The dynamics were then repeated in an interleaved manner until 16 were acquired at each frequency, making a total of 32 dynamics. In all scans, the repetition time (TR) was 2000 ms with 1024 samples. The voxel size of 20 × 20 × 20 mm^3^ was chosen in the middle of the phantom. Receiver bandwidth was 2000 Hz, water suppression method had an excitation with a window of 140 Hz, and the shimming was second‐order pencil beam. The excitation pulse bandwidth was 1987 Hz and the refocusing pulse bandwidth was 1263 Hz. The same water‐suppression and shimming techniques, as well as the same values for receiver bandwidth, pulse bandwidths, and number of samples, were used in the other measurements. The Ace peak in the spectra was used for frequency referencing at 1.92 ppm. This phantom experiment was also simulated using NMRSCOPE 31, a jMRUI 32 routine in which coupling constants and chemical shift values are automatically set based on Govindaraju et al 6. Time‐domain metabolite signals were generated quantum‐mechanically under the pulse sequence conditions used in vitro. TE was varied from 30–250 ms. The simulation results were corrected for T_2_ relaxation effects.

### Determining the Accuracy of GSH Measurements from PRESS and MEGA‐PRESS

#### Brain‐Mimicking Phantoms

Twelve phantom solutions with a range of GSH concentrations (0, 0.5, 0.75, 1, 1.5, 2, 2.5, 3, 4, 8, 12, 24 mM) were prepared in phosphate buffer (25 mM KH_2_PO_4_; 25 mM K_2_HPO_4_) and adjusted to pH 7.0. The phantoms contained brain metabolites that interfere with the GSH signal, at the following physiological concentrations 6: N‐acetylaspartate (NAA, 12.25 mM), creatine (Cr, 7.85 mM), choline (Cho, 1.7 mM), glutamate (Glu, 9.25 mM), glutamine (Gln, 4.4 mM), myo‐Inositol (myo‐Ins, 5.95 mM), aspartate (Asp, 1.2 mM), and γ‐aminobutyric acid (GABA, 1.6 mM). All chemicals were purchased from Sigma‐Aldrich (Dorset, United Kingdom). Phantom temperature was kept at 21 ºC for data acquisition. PRESS spectra were acquired with TE = 35 ms, TR = 2000 ms, 64 averages per dynamic, four dynamics with 1024 samples and voxel size = 30 × 30 × 30 mm^3^. An additional water‐reference scan (four averages, no water suppression) was acquired, which was used as a concentration reference and to automatically phase the spectrum. MEGA‐PRESS spectra (TE = 130 ms, TR = 2000 ms, four averages per dynamic, 32 dynamics for each MEGA pulse frequency for a total of 64 dynamics, voxel size = 30 × 30 × 30 mm^3^) with a Gaussian selective pulse duration of 14 ms and bandwidth of 106 Hz were acquired from each phantom. The total number of averages and the acquisition time were the same for both sequences. Shorter but more numerous dynamics were used for MEGA‐PRESS to provide the opportunity of correcting dynamic to dynamic frequency and phase variations 33. However, both in vivo and in vitro, the high stability of the scanner, and the full cooperation of the healthy volunteers made the corrections unnecessary.

#### In Vivo

**Figure 1:**
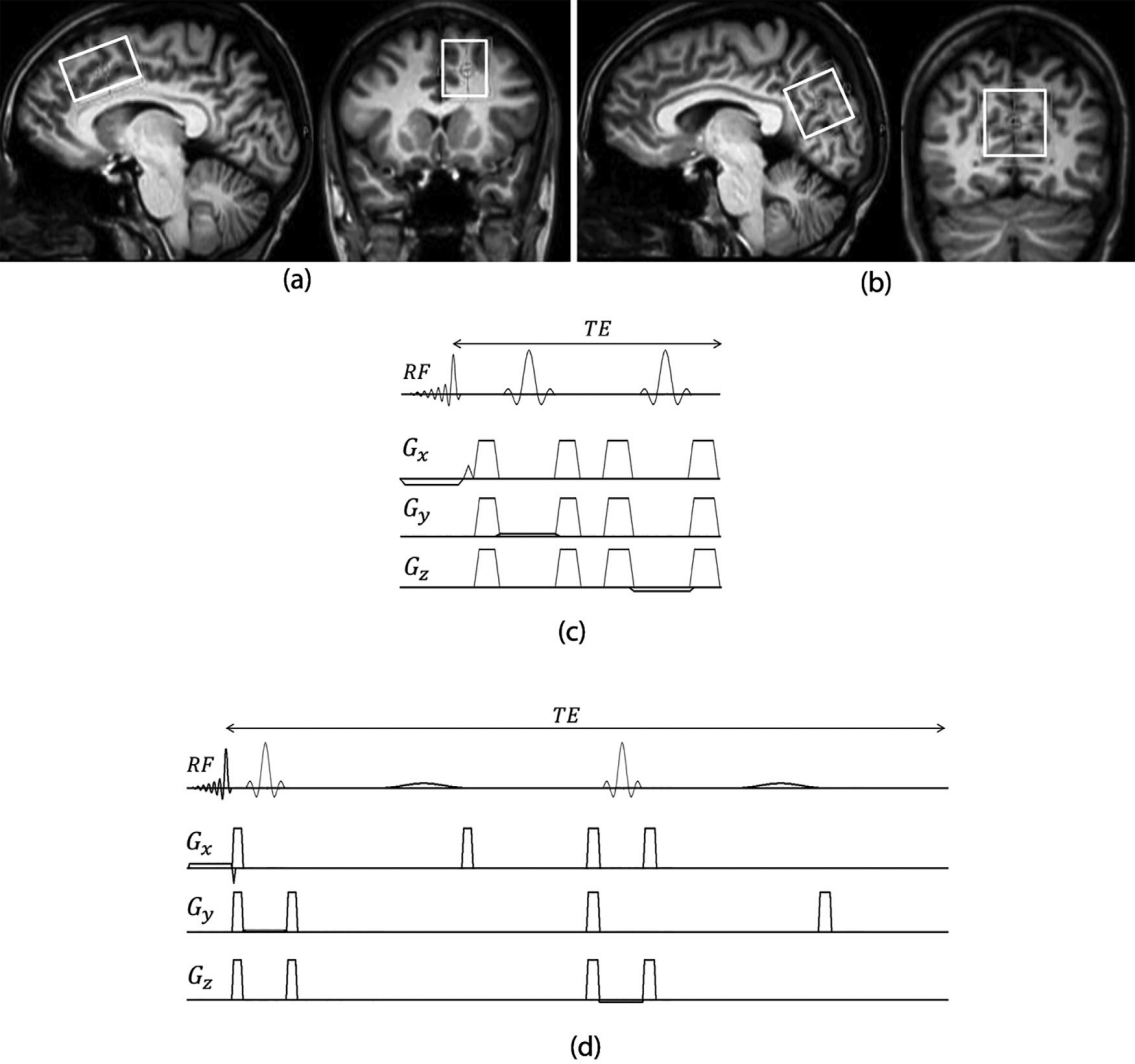
T_1_ images showing the positioning of the ^1^H MRS voxel in the anterior cingulate cortex (a) and the occipital cortex (b). Diagrams for the PRESS (c) and MEGA‐PRESS (d) pulse sequences used in this study.

Spectra were acquired from seven healthy volunteers (five females, two males, age range 23–35 years old) who all gave informed consent in accordance with procedures approved by the local ethics committee. A total of five measurements in two different brain regions were made on each volunteer as follows: A voxel sized 40 × 25 × 25 mm^3^ was placed in the left anterior cingulate cortex (ACC) (see Fig. 1). A TE = 35 ms PRESS spectrum was acquired with TR = 2000 ms, two dynamics, and 128 averages per dynamic. An additional water reference scan (four averages, no water suppression) was acquired, which was used as a concentration reference and to automatically phase the spectrum. A MEGA‐PRESS spectrum with TE = 130 ms, TR = 2000 ms, 64 dynamics, and four averages per dynamic with a Gaussian‐selective pulse duration of 14 ms and bandwidth of 106 Hz was also acquired in the same voxel. To confirm in vivo that TE = 130 ms yields more signal than TE = 70 ms for MEGA‐PRESS, an additional MEGA‐PRESS sequence with TE = 70 ms was run in the ACC voxel. A volume of 30 × 30 × 30 mm^3^ was placed in the occipital cortex (OCC) (see Fig. 1), and the MEGA‐PRESS TE = 130 ms and the PRESS TE = 35 ms were repeated as in the ACC voxel.

### MRS Quantification

There were two types of spectra in this study: PRESS and MEGA‐PRESS. All spectroscopy processing and CRLB calculation, both in vitro and in vivo, was done using routines in jMRUI software package 32. A nonlinear least‐squares fitting algorithm was used for both data types. The PRESS spectra required detailed prior knowledge, while the simplicity of the MEGA‐PRESS spectra made this unnecessary. Hence, two different tools were used for quantification of the spectra. The detailed quantification method is described next.

#### PRESS Data

Analysis of the spectroscopic data was performed using QUEST, a time domain algorithm that fits a weighted combination of metabolite signals directly to the data acquired in vivo 7. An initial metabolite basis set for the appropriate sequence timings was obtained using the routine NMRSCOPE, in which the theoretical signals of metabolites can be computed by quantum mechanics based on the modified product‐operator formalism 31. The following metabolite signals were simulated for the human data set: NAA, Cr, Cho, Asp, GABA, glucose, Glu, Gln, myo‐Ins, scyllo‐Inositol, and GSH. For analysis of the phantom data, the basis set included only the metabolites present in the phantom: NAA, Cr, Cho, Glu, Gln, myo‐Ins, Asp, GABA, and GSH.

Spectra were phased prior to analysis using the Ace (1.92 ppm) or NAA (2.02 ppm) peak as reference. Residual water was removed using the Hankel Lanczos singular values decomposition (HLSVD) routine in jMRUI. The macromolecular background signal was estimated using the Subtract routine in QUEST from the first 12 points in the FID. The unsuppressed water signal was used as internal reference for quantification.

#### MEGA‐PRESS Data

Analysis of the spectroscopic data was performed using AMARES, a peak‐by‐peak quantification method based on a nonlinear least‐squares quantitation algorithm that requires starting values for the parameters to be estimated 8. The starting values are singlets set at appropriate frequency shifts with a line‐shape definition of either Lorentzian or Gaussian. For accurate frequency referencing and phase estimation of the spectrum, preprocessing was performed as follows: The dynamics that had their MEGA frequency set at 4.56 ppm (MEGA‐on spectra) were summed. The NAA peak was set as 2.02 ppm and its phase was estimated using AMARES. This phase was applied to all dynamics (MEGA‐on and MEGA‐off). In this implementation of MEGA‐PRESS on the Philips scanner 23, there was an extra 180 º phase shift for the MEGA‐off acquisition, so the final MEGA‐PRESS spectrum was the sum of the MEGA‐on and MEGA‐off dynamics. Any residual water peak was removed using the HLSVD filter. GSH, NAA, and Cr were quantified using defined prior knowledge, including the frequency shift and the Lorentzian line shape. Because the phase estimation was done in the preprocessing step, the phase in the quantification process was set to the calculated value. The reference water signal from the PRESS acquisition was used as a concentration reference.

#### Editing Efficiency

In J‐edited spectra, because the bandwidth of the slice‐selective pulses is similar to the chemical shift difference of the GSH‐coupled spin system, the evolution of the scalar coupling of C7‐GSH protons becomes spatially dependent, resulting in signal loss 22, 34, 35. Hence, to have a proper comparison of the PRESS and the MEGA‐PRESS signal, a calculation of the MEGA‐PRESS signal loss has been made. A MEGA‐PRESS difference‐edited spectrum is obtained by subtracting N/2“MEGA‐on” scans ( $S_{MEGA-on})$ from N/2“MEGA‐off” scans ( $S_{MEGA-off})$, so the editing efficiency ( eff) can be calculated as follows:

$$eff=\frac{S_{edited}}{S_{available\;}}=\frac{\sum_{N}(S_{MEGA-on}-S_{MEGA-off})}{2\times \sum_{2}S_{MEGA-off}}$$

where $S_{edited}$ is the edited MEGA‐PRESS signal acquired from the scanner, and $S_{available\;}$ is the signal available when the editing pulses are turned off. The factor of 2 compensates for the fact that the edited signal is from twice as many averages as the MEGA‐off signal. To calculate the editing efficiency of GSH, the GSH/Ace phantom data were used (determined to be 0.74 at TE = 130 ms).

#### Concentration Calculation

Water‐referenced GSH concentrations were estimated using the following equation 22:

$$\left[GSH\right]=\frac{S_{GSH}}{S_{H_{2}O}}\times \left[H_{2}O\right]\times VIS_{H_{2}O}\times \frac{1-\text{exp}\left(-\frac{TR}{T_{1H_{2}O}}\right)}{1-\text{exp}\left(-\frac{TR}{T_{1GSH}}\right)}\times \frac{\text{exp}\left(-\frac{TE}{T_{2H_{2}O}}\right)}{\text{exp}\left(-\frac{TE}{T_{2GSH}}\right)}\times \frac{1}{eff}$$

where $S_{GSH}$ and $S_{H_{2}O}$ are the raw signals of GSH and water, respectively; $\left[H_{2}O\right]$ is the concentration of pure water (110 mM, the default value in jMRUI ); $VIS_{H_{2}O}$ is the percentage of water in brain tissue with the value of 0.8; eff is the editing efficiency (determined as previously to be 0.74); $T_{1H_{2}O}$ and $T_{2H_{2}O}$ are the T_1_and T_2_ of water, respectively; and $T_{1GSH}$ and $T_{2GSH}$ are the T_1_ and T_2_ of GSH, respectively.

The in vitro T_1_ was estimated from the GSH/Ace phantom using saturation recovery data: a PRESS pulse sequence with a fixed TE = 35 ms and a variable TR with six different values from 2 to 17 s. $T_{1GSH}=350$ ms and $T_{1H_{2}O}=4400$ ms estimates were obtained from the nonlinear least‐squares fit of the amplitude of the fitted time domain signal, which is equivalent to the area under the peak in the frequency domain (signal intensity) measured at each TR value. For measuring in vitro $T_{2}$, a PRESS pulse sequence with a fixed TR = 2000 ms and a variable TE with 15 different values from 70 to 240 ms was carried out. $T_{2GSH}=150$ ms and $T_{2H_{2}O}=2800$ ms estimates were obtained from the nonlinear least‐squares fit of the signal intensity measured at each TE value. The relaxation values of water in vivo were assumed to be $T_{1H_{2}O}=1100\;ms$ and $T_{2H_{2}O}=95\;ms$ 22, and the GSH values were assumed to be $T_{1GSH}=400$ ms 36 and $T_{2GSH}=67$ ms 37.

#### Segmentation

The $T_{1}$‐weighted images, acquired to position the MRS voxels during the scan, were segmented with a statistical parametric mapping approach using spm8 (http://www.fil.ion.ucl.ac.uk/spm/). Voxel registration was performed using custom‐made scripts developed in MATLAB (The MathWorks, Natick, Massachusetts, USA) by Dr. Nia Goulden, which can be accessed at http://biu.bangor.ac.uk/projects.php.en. The scripts generated a mask for voxel location by combining location information from the Philips SPAR file with orientation and location information contained within the T_1_ image. The application of this mask to the gray matter (GM), white matter (WM), and cerebrospinal fluid (CSF) images enabled the calculation of partial volume by establishing the percentage of each tissue type within the relevant voxels. These percentages were used to correct metabolite concentrations for differences in cerebrospinal fluid content (assumed to contain no GSH).

#### Statistical Analysis

Paired two‐tailed t‐tests were performed on GSH concentrations estimated from PRESS and MEGA‐PRESS spectra in each voxel of the human brain. The hypothesis was that if GSH could be accurately quantified from PRESS, there should not be a significant difference in the concentrations calculated from PRESS and MEGA‐PRESS in each region. Paired t‐tests were also used to assess whether the GSH content was different between the two voxels when estimated from the same type of spectra (either both from PRESS or both from MEGA‐PRESS), and to determine whether the white and gray matter contents were significantly different between the two voxels. Statistical significance was considered reached when *P* < 0.05.

## RESULTS

### TE Optimization

#### Simulation and GSH/Ace Phantom

**Figure 2:**
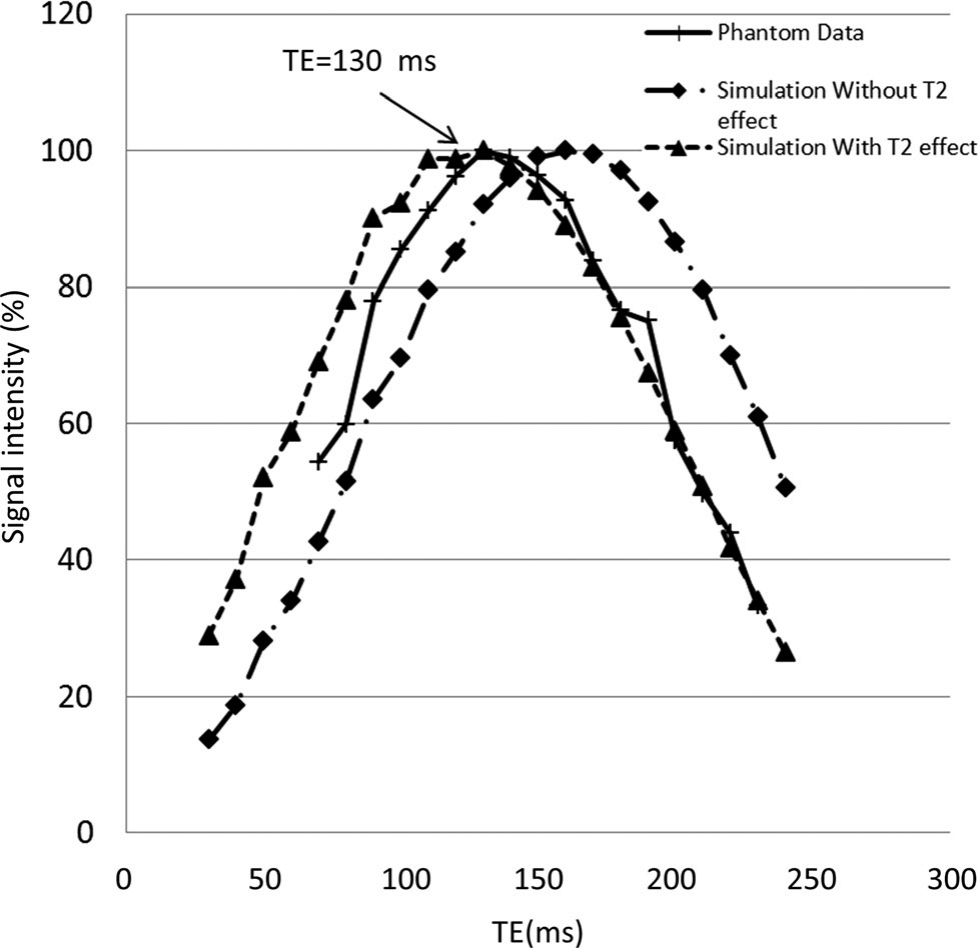
Simulation and phantom signal intensity results of the MEGA‐PRESS pulse sequence showing the maximum signal at TE = 130 ms.

**Figure 3:**
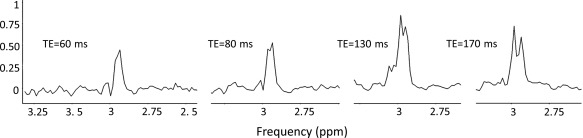
GSH signal from the GSH/Ace phantom using the MEGA‐PRESS pulse sequence at different TE values.

Figure 2 illustrates curves of GSH signal versus TE. There are two simulation curves, one taking the T_2_ effect into account, and the other without this correction. The simulation results are overlaid with the GSH signal from the GSH/Ace phantom. The simulation with the T_2_ relaxation and the measurement in vitro were in good agreement; the maximum signal was acquired at TE = 130 ms (see Fig. 3 for edited spectra at varying TEs).

#### In Vivo

**Figure 4:**
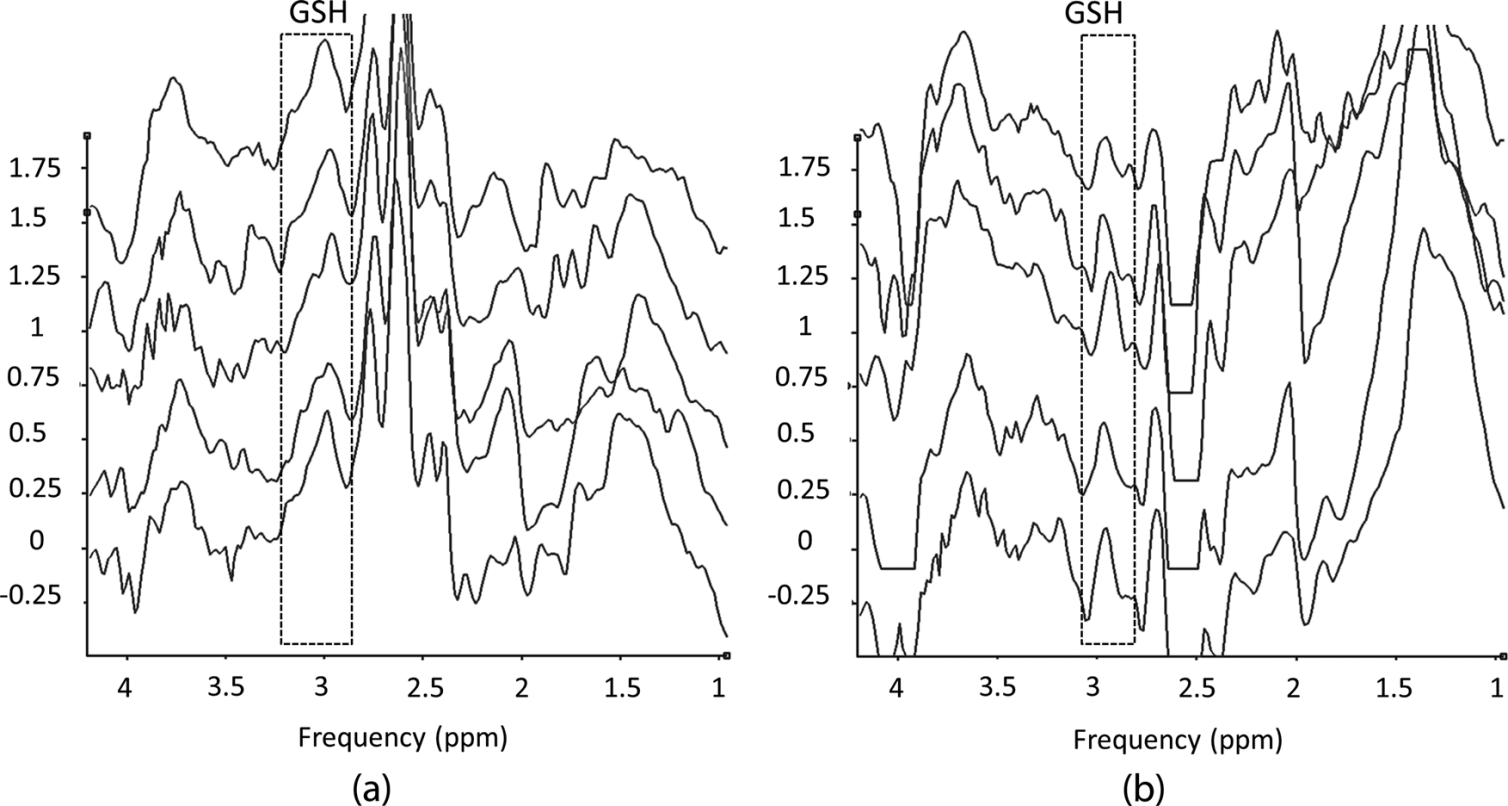
GSH signals from an ACC voxel in five different healthy volunteers, acquired with TE = 130 MEGA‐PRESS (a) and TE = 70 MEGA‐PRESS (b).

The GSH concentration [mean ± standard deviation (SD)] was calculated to be 3.2 ± 0.6 mM at TE = 130 ms and 2.0 ± 1.1 mM at TE = 70 ms from MEGA‐PRESS acquisitions. The average CRLB at TE = 130 ms was 18 ± 5% and 26 ± 5% at TE = 70 ms. The average ratio of GSH signal at TE = 130 ms over TE = 70 ms was 1.8 ± 0.6 (Fig. 4). No significant correlation was found between the two TE acquisitions.

### MEGA‐PRESS and PRESS Comparison

#### Brain‐Mimicking Phantoms

**Figure 5:**
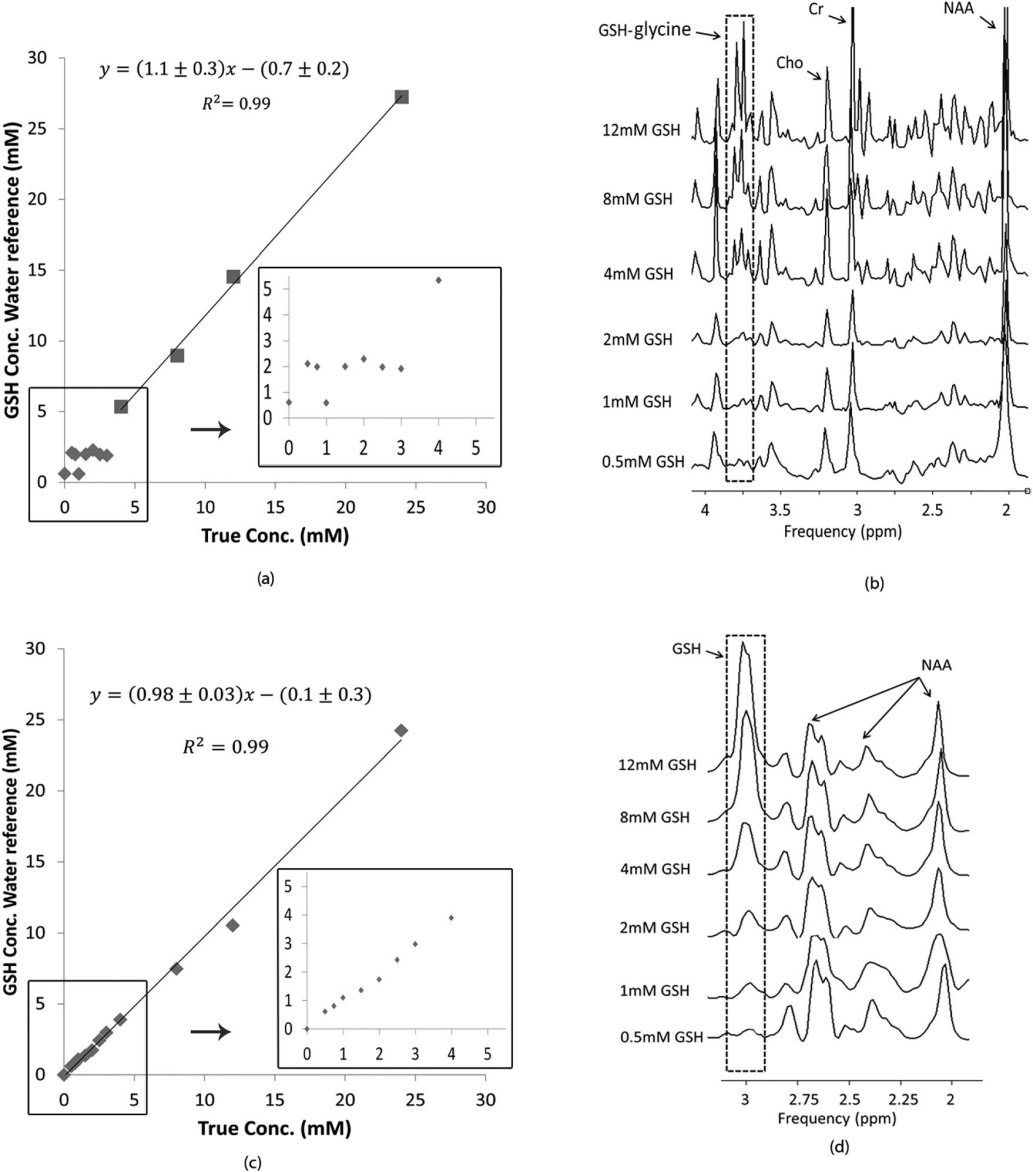
GSH concentration quantified in phantoms versus the actual concentration in spectra acquired using PRESS (a) and the spectra of six phantoms with different GSH concentrations (b). The corresponding MEGA‐PRESS data can be seen in (c) and (d). The zoomed‐in regions show that, despite the strong linear relationship for PRESS at high concentrations, the relationship is chaotic at physiological concentrations. In contrast, MEGA‐PRESS is well‐behaved in both locations.

The results from the brain‐mimicking phantom experiments are presented in Table 1 and Figure 5. When using QUEST to quantify GSH from PRESS spectra, it was not possible to discriminate GSH concentrations within the physiological range (<4 mM). In this concentration range a similar concentration for GSH was returned from PRESS, regardless of its actual concentration (Fig. 5 and Table 1). GSH could only be quantified accurately from PRESS spectra if present at high concentrations (≥4 mM). The CRLBs of the quantified GSH in all phantoms were below 28%. On the contrary, the GSH concentration was accurately quantified from the MEGA‐PRESS acquisitions even down to the lowest concentration of 0.5 mM (Fig. 5). As illustrated in Figure 5, the PRESS linear fit for concentrations of 4 mM and higher does not go through the origin and the slope is 1.1, whereas in estimations from MEGA‐PRESS the slope is ∼1 and the linear fit goes through the origin. Table 1 presents the estimated and true concentrations and CRLBs for all of the phantoms.

**Table 1:** GSH Concentrations Measured from Phantoms in PRESS and MEGA‐PRESS TE = 130 ms Spectra (in mM)

		PRESS estimate		MEGA‐PRESS estimate	
Phantom	True GSH	GSH	CRLB % of estimate	GSH	CRLB % of estimate
1	0	0.62	14%	0	—
2	0.5	2.11	18%	0.60	17%
3	0.75	2.00	15%	0.81	19%
4	1	0.68	17%	1.09	32%
5	1.5	2.01	18%	1.36	21%
6	2	2.30	27%	1.73	22%
7	2.5	1.99	16%	2.43	15%
8	3	1.9	15%	2.98	12%
9	4	5.34	15%	3.90	9.6%
10	8	8.96	10%	7.48	2%
11	12	14.50	9%	10.54	2%
12	24	27.2	5%	24.25	1.6%

#### In Vivo

**Figure 6:**
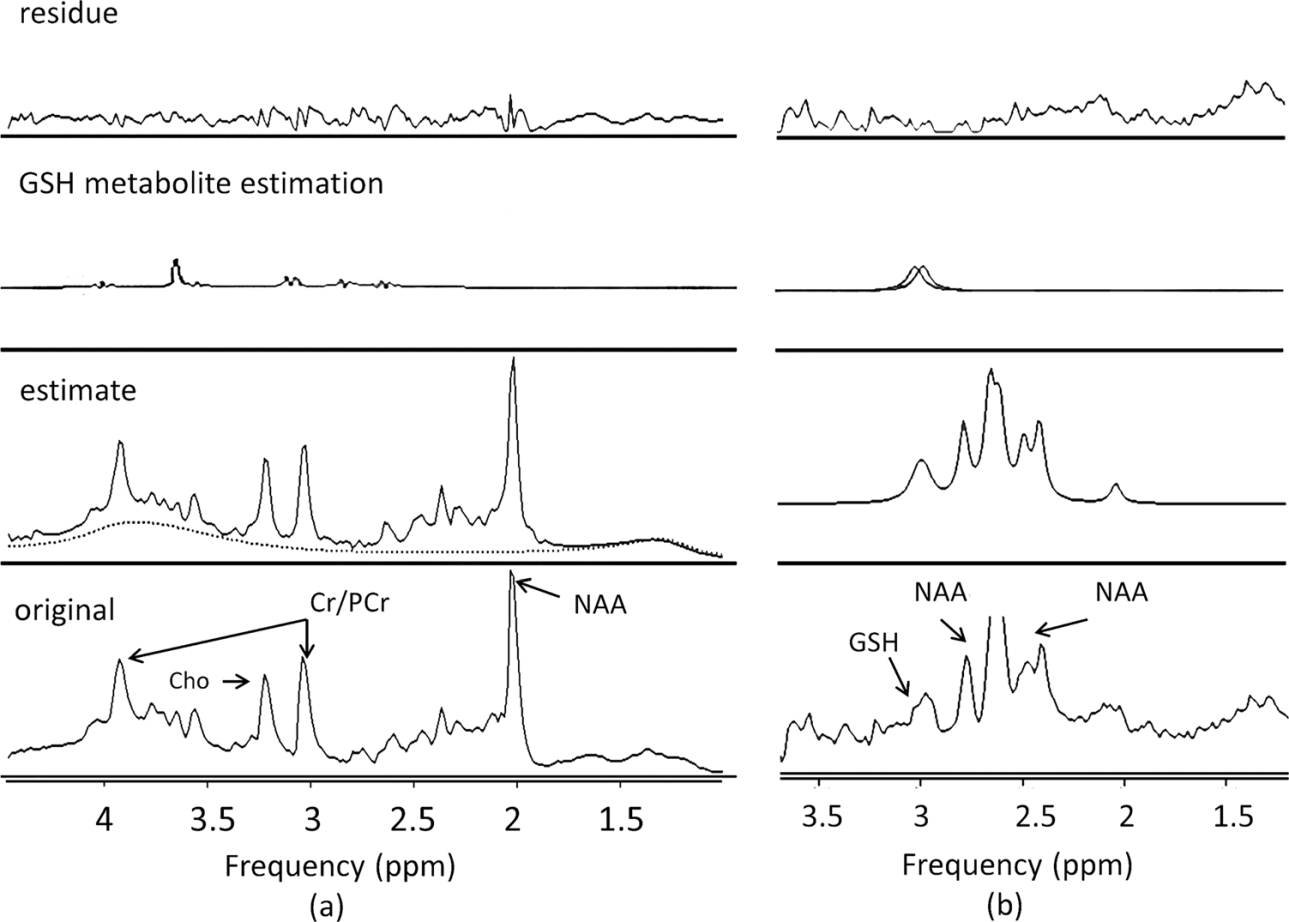
ACC voxel spectra and fitting with jMRUI, using PRESS (a) and MEGA‐PRESS TE = 130 ms (b) acquisition.

In the PRESS spectra, no particular peak can be assigned to GSH, whereas the GSH signal can be clearly identified in the MEGA‐PRESS spectra (Fig. 6). Table 2 presents the mean ± SD of the GSH concentrations and CRLBs calculated from PRESS and MEGA‐PRESS spectra. GSH concentrations quantified from PRESS were not significantly different between the two regions (*P* = 0.4). Figure 6 illustrates the quantification of a healthy volunteer PRESS and MEGA‐PRESS spectrum. GSH was significantly higher in ACC compared with OCC when quantified from the MEGA‐PRESS spectra (*P* = 0.003). There was no significant difference between PRESS and MEGA‐PRESS GSH estimations in the ACC (*P* = 0.3), whereas there was a significant difference in the OCC (*P* = 0.003).

**Table 2:** Average GSH Concentrations Quantified from In Vivo PRESS and MEGA‐PRESS TE = 130 ms Spectra (in mM)

Voxel position	PRESS estimate		MEGA‐PRESS estimate	
	GSH ± SD	CRLB ± SD	GSH ± SD	CRLB ± SD
ACC	2.8 ± 0.3	19 ± 3%	3.2 ± 0.6	18 ± 5%
OCC	2.5 ± 0.7	12 ± 2%	1.4 ± 0.4	24 ± 6%

#### Segmentation Results

**Figure 7:**
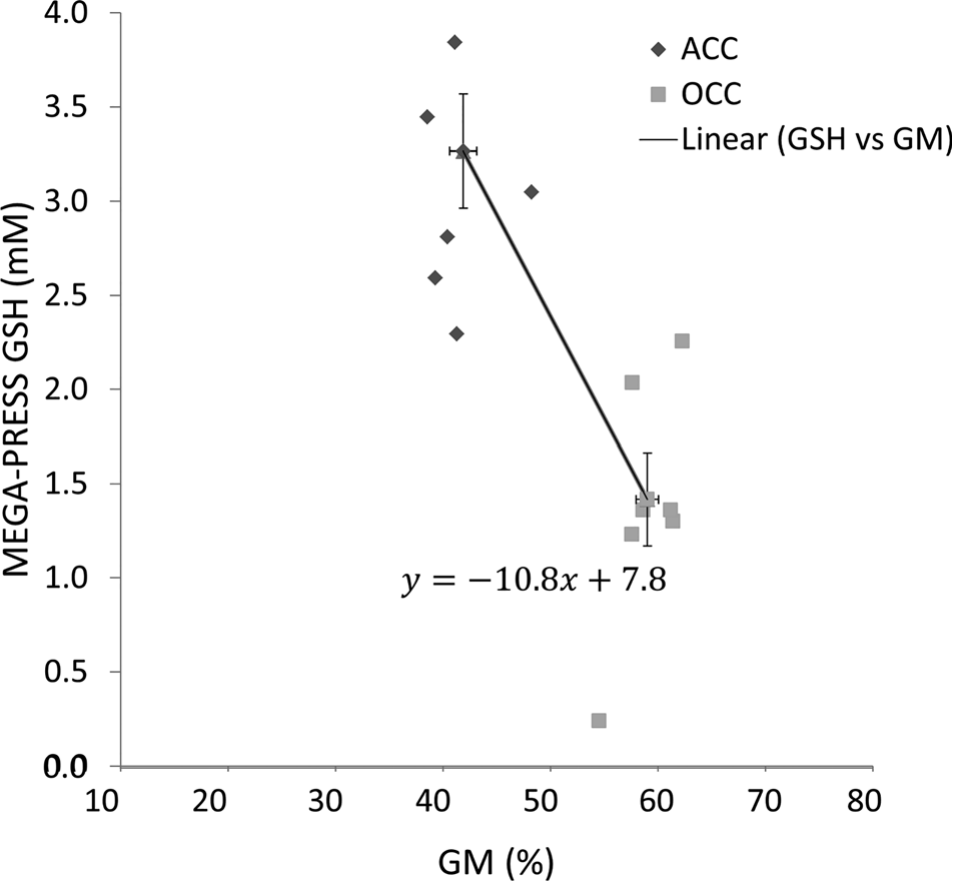
Mean of GM percentages versus mean of GSH estimations from TE = 130 MEGA‐PRESS acquisitions. The standard errors of mean are plotted as error bars. Individual values for each subject are also plotted.

The percentages (mean ± SD) of GM, WM, and CSF in ACC were 41.9 ± 3.3%, 50.8 ± 5.0%, and 7.3 ± 2.0%, respectively. The corresponding segmentation percentages in OCC were 59.0 ± 2.7%, 28.7 ± 3.8%, and 12.3 ± 2.2%, respectively. A two‐tailed paired t‐test detected that GM (*P* < 0.0005) and CSF (*P* < 0.005) contents (in percentages) were significantly higher in the OCC MRS voxel compared with the ACC voxel, whereas the content of WM was significantly lower in the OCC voxel compared with the ACC voxel (*P* < 0.0005). The average GM proportion versus the average GSH concentration estimated from MEGA‐PRESS is illustrated for each of the ACC and OCC voxels in Figure 7.

## DISCUSSION AND CONCLUSIONS

The simulation results that include the effect of T_2_ relaxation were in good agreement with the GSH/Ace phantom data (Fig. 2), confirming that TE = 130 ms is optimal for detection of GSH by MEGA‐PRESS, due to higher editing efficiency compared with the commonly used TE ∼70 ms 10, 11, 18. Our finding is in line with two other studies also presenting TE ∼130 ms (TE = 131 ms 29, TE = 120 ms 39) as the optimum for detecting GSH using MEGA‐PRESS. However, the optimum TE will depend on field strength and temperature 29. In vivo, the average GSH signal intensity acquired from the ACC of seven healthy volunteers at TE = 130 ms was 1.8 ± 0.6‐fold higher than at TE = 70 ms (Fig. 4), confirming the findings of the GSH/Ace phantom study. Moreover, the signal‐to‐noise ratio and the CRLB both showed a better signal quality at TE = 130 ms compared with TE = 70 ms. There was no significant correlation found between the concentration estimated from the spectra at TE = 130 ms and TE = 70 ms. This could be the result of relatively low intersubject variability or low signal to noise at TE = 70 ms.

When fitting the PRESS spectra acquired in the brain‐mimicking phantoms, it is clear that at physiological concentrations (<4 mM) the estimates are imprecise and inaccurate (Fig. 5 and Table 1). This undermines the contention that physiological levels of GSH can be accurately and reliably measured from PRESS TE = 35 ms. Additionally, our data from the GSH/Ace phantom showed that the glycine component of GSH was a pseudo‐doublet, supporting the interpretation of Kaiser et al 10 and suggesting that simulations of GSH using just the coupling and chemical shifts from Govindaraju et al 6, which consist of prior knowledge in most quantification software, cannot fully model the glycine proton of its spectrum. This can add further inaccuracy to the quantification of GSH using PRESS spectra. In contrast, the GSH glycine peak cancels out using MEGA‐PRESS editing at 4.56 and 1.44 ppm.

The inaccurate detection and estimation of GSH from PRESS could not have been revealed by CRLB values, as they were below 28% in all measurements. This observation is in agreement with Kreis 38, who suggested that CRLB cannot be used as a sole criterion to assess quantification reliability for MRS data. However, the low CRLBs (less than 10%) in high GSH concentrations in the brain‐mimicking phantom study can be a further assurance that GSH in high concentrations ( ≥ 4 mM) can be accurately quantified in PRESS spectra.

This study proved that when fitting PRESS spectra, the solution for GSH concentration found by the mathematical algorithm may be consistent and within the expected range, but can still be inaccurate. Other studies 12, 13, 40 that analyzed the relationship between GSH estimations from PRESS or STEAM pulse sequences at 3 T and known values reported a linear relationship between true and estimated GSH concentrations. However, inspection of the data presented shows that MRS estimations of GSH are nonzero for phantoms without GSH, and the values returned for all concentrations lower than 3 mM are similar. Moreover, the linear fit from which physiological concentration values are derived is driven by the values from the phantoms containing high concentrations of GSH. It is difficult to assess the accuracy of the method at low concentrations because of too few data points.

In humans, the concentrations calculated from the PRESS and MEGA‐PRESS spectra were significantly different in the OCC region. In addition, results from MEGA‐PRESS, but not from PRESS, detected significantly different concentrations between the two regions. One reason for differing concentrations could be a different GM/WM ratio, with GM usually presumed to have higher metabolite concentrations, as found by 27. However, we found higher GM content in OCC, which had a lower GSH concentration than ACC. Additionally, if we extrapolate the line on Figure 7, we could see that GSH would have a 0 mM concentration for 70% GM content, which is unrealistic. This suggests that the significant difference in GSH content between the two voxels is rather a genuine, physiological feature based on regional concentration differences rather than a result of differences in GM and WM contents. The literature also suggests a high to low GSH concentration gradient in frontal to occipital brain regions 2, 3, 27. Interestingly, although MEGA‐PRESS results proved to be more reliable, the CRLB values from PRESS spectra fitting were lower than the MEGA‐PRESS ones. In addition to the brain‐mimicking phantom results, this also challenges the infallibility of CRLB values as being the sole criterion for assessing quantification reliability, as discussed in Kreis 38. Without ground truth on ACC and OCC GSH concentrations, we cannot say with certainty that MEGA‐PRESS measurements are correct and those using PRESS are not. Nevertheless, when taking into account the brain‐mimicking phantom data, the likelihood of a positive detection of a regional GSH concentration difference by MEGA‐PRESS is higher than the lack of detection of regional differences from PRESS. The data acquired in this study can be available on request for further investigation on the fitting methods.

In summary, because of the uncertainties in GSH quantification raised by the phantom and human study, we conclude that normal physiological concentrations of GSH cannot be reliably quantified using PRESS at 3 T, whereas in MEGA‐PRESS the GSH signal is visually detectable and more accurate quantification can be performed. Therefore, we recommend using editing pulse sequences rather than PRESS TE = 35 ms; however, this may increase the scanning time if nonedited acquisitions are also needed for quantification of other metabolites. These results support the case for using MEGA‐PRESS GSH editing sequence with TE = 130 ms in future studies aiming at assessing possible GSH concentration alterations in psychiatric and other brain diseases in which the oxidative equilibrium could be disturbed.
